# Butyrate Levels in the Transition from an Infant- to an Adult-Like Gut Microbiota Correlate with Bacterial Networks Associated with *Eubacterium Rectale* and *Ruminococcus Gnavus*

**DOI:** 10.3390/genes11111245

**Published:** 2020-10-22

**Authors:** Morten Nilsen, Carina Madelen Saunders, Inga Leena Angell, Magnus Ø. Arntzen, Karin C. Lødrup Carlsen, Kai-Håkon Carlsen, Guttorm Haugen, Live Heldal Hagen, Monica H. Carlsen, Gunilla Hedlin, Christine Monceyron Jonassen, Björn Nordlund, Eva Maria Rehbinder, Håvard O. Skjerven, Lars Snipen, Anne Cathrine Staff, Riyas Vettukattil, Knut Rudi

**Affiliations:** 1 Faculty of Chemistry, Biotechnology and Food Science, Norwegian University of Life Sciences, 1430 Ås, Norway; inga.angell@nmbu.no (I.L.A.); magnus.arntzen@nmbu.no (M.Ø.A.); live.hagen@nmbu.no (L.H.H.); christine.monceyron.jonassen@so-hf.no (C.M.J.); lars.snipen@nmbu.no (L.S.); knut.rudi@nmbu.no (K.R.); 2 Division of Paediatric and Adolescent Medicine, Oslo University Hospital, 0450 Oslo, Norway; k.c.l.carlsen@medisin.uio.no (K.C.L.C.); k.h.carlsen@medisin.uio.no (K.-H.C.); h.o.skjerven@medisin.uio.no (H.O.S.); m.r.vettukattil@medisin.uio.no (R.V.); 3 Faculty of Medicine, Institute of Clinical Medicine, University of Oslo, 0318 Oslo, Norway; guttorm.haugen@medisin.uio.no (G.H.); e.m.rehbinder@medisin.uio.no (E.M.R.); a.c.staff@medisin.uio.no (A.C.S.); 4 Division of Obstetrics and Gynaecology, Oslo University Hospital, 0450 Oslo, Norway; 5 Department of Nutrition, Faculty of Medicine, Institute of Basic Medical Sciences, University of Oslo, 0405 Oslo, Norway; m.h.carlsen@medisin.uio.no; 6 Astrid Lindgren Children’s Hospital, Karolinska University Hospital, 17176 Stockholm, Sweden; gunilla.hedlin@ki.se (G.H.); bjorn.nordlund@ki.se (B.N.); 7 Department of Women’s and Children’s Health, Karolinska Institutet, 17176 Stockholm, Sweden; 8 Genetic Unit, Centre for Laboratory Medicine, Østfold Hospital Trust, 1714 Kalnes, Norway; 9 Department of Dermatology, Oslo University Hospital, 0424 Oslo, Norway

**Keywords:** gut microbiota, infant, short-chain fatty acids, metaproteomics

## Abstract

Relatively little is known about the ecological forces shaping the gut microbiota composition during infancy. Therefore, the objective of the present study was to identify the nutrient utilization- and short-chain fatty acid (SCFA) production potential of gut microbes in infants during the first year of life. Stool samples were obtained from mothers at 18 weeks of pregnancy and from infants at birth (first stool) at 3, 6, and 12-months of age from the general population-based PreventADALL cohort. We identified the taxonomic and SCFA composition in 100 mother-child pairs. The SCFA production and substrate utilization potential of gut microbes were observed by multiomics (shotgun sequencing and proteomics) on six infants. We found a four-fold increase in relative butyrate levels from 6 to 12 months of infant age. The increase was correlated to *Eubacterium rectale* and its bacterial network, and *Faecalibacterium prausnitzii* relative abundance, while low butyrate at 12 months was correlated to *Ruminococcus gnavus* and its associated network of bacteria. Both *E. rectale* and *F. prausnitzii* expressed enzymes needed for butyrate production and enzymes related to dietary fiber degradation, while *R. gnavus* expressed mucus-, fucose, and human milk oligosaccharides (HMO)-related degradation enzymes. Therefore, we believe that the presence of *E. rectale,* its network, and *F. prausnitzii* are key bacteria in the transition from an infant- to an adult-like gut microbiota with respect to butyrate production. Our results indicate that the transition from an infant- to an adult-like gut microbiota with respect to butyrate producing bacteria, occurs between 6 and 12 months of infant age. The bacteria associated with the increased butyrate ratio/levels were *E. rectale* and *F. prausnitzii*, which potentially utilize a variety of dietary fibers based on the glycoside hydrolases (GHs) expressed. *R. gnavus* with a negative association to butyrate potentially utilizes mucin, fucose, and HMO components. This knowledge could have future importance in understanding how microbial metabolites can impact infant health and development.

## 1. Introduction

The temporal development of the gut microbiota during infancy is essential for immunological and developmental programming [1]. Although there has been some debate on whether colonization happens before birth, based on bacterial findings in placentas [2], umbilical cords [3], and meconium [4], recent studies, including our own [5], have challenged this view in support of the sterile womb hypothesis [5,6]. Regardless of when colonization occurs, the bacterial diversity in vaginally delivered newborns largely represent bacteria from the mother’s natural vaginal flora, such as *Lactobacillus*, *Bifidobacterium*, *Prevotella,* and *Sneathia* [5,7], as well as from the maternal gut flora [8]. The earliest colonization often consists of Proteobacteria, which is likely involved in facilitating a suitable environment for anaerobic bacteria by depleting the gut of oxygen [7]. The oxygen depletion increases the amount of *Bacteroides*, *Clostridium,* and *Bifidobacterium*. *Bifidobacterium* dominates from an early stage until weaning, due to their capacity to break down human milk oligosaccharides [7,9,10]. After weaning, the infant’s gut microbiota composition starts to resemble the adult gut microbiota in terms of anaerobes, such as *Clostridium* and *Bacteroides* [11].

A vital group of metabolites are the short-chain fatty acids (SCFAs), which are produced by gut microbes and which influence immune modulation, control anabolic processes, serve as an energy source for colonocytes, and are precursor metabolites for lipogenesis and gluconeogenesis [12,13]. Butyrate is essential in regulating immune responses by controlling inflammation responses and serving as the main energy source for gut epithelial cells [14,15]. Although the importance of SCFAs has been well established, we lack detailed knowledge about their longitudinal development in the first year of life and the bacteria responsible for their production.

The objective of the present study was to identify the nutrient utilization and the SCFA production potential of gut microbes in infants during the first year of life. First, we characterized the taxonomy of gut bacteria in pregnant women and their infants through the first 12 months of life. We then identified SCFA composition in pregnant women and their infants through the first year of life and correlated the SCFA composition with gut bacteria and influencing factors. Finally, we assessed the mechanism for potential polysaccharide utilization and SCFA production potential through metaproteomics.

## 2. Materials and Methods

We analyzed samples from the general population-based cohort, the Preventing Atopic Dermatitis and ALLergy (PreventADALL) study [16] that included 2397 mother-child pairs from Norway and Sweden. The primary aim of PreventADALL was to prevent allergic disease development, and secondarily to assess early life factors involved in the development of non-communicable diseases. Fecal samples from this study were collected at the enrollment of the mother at approximately 18 weeks of pregnancy, and in the infant at birth (meconium), 3, 6, and 12 months of age. In the present study, we used a multiomics approach to analyze fecal samples in the first 100 mother-child pairs that had infant stool samples available from at least three out of four sampling time points. The included children, of whom 51 were boys, were born at 39.6 ± 1.6 weeks of gestation with a birthweight of 3577 ± 529 g. A total of 22 infants were delivered via caesarean section and 78 vaginally. Eighty-three infants were breastfed between 3 and 6 months (nine missing), 73 infants between 6 and 9 months (four missing), and 49 between 9 and 12 months (10 missing).

Informed written consent from all pregnant mothers was received upon inclusion, and from both parents upon inclusion of the newborn child. The PreventADALL study has been approved by the Regional Ethical Committee (REK) for Medical and Health Research Ethics in South-Eastern Norway (2014/518) as well as in Sweden (2015/4:3) by the Regional Ethical Trial Committee of Stockholm.

**Figure 1:**
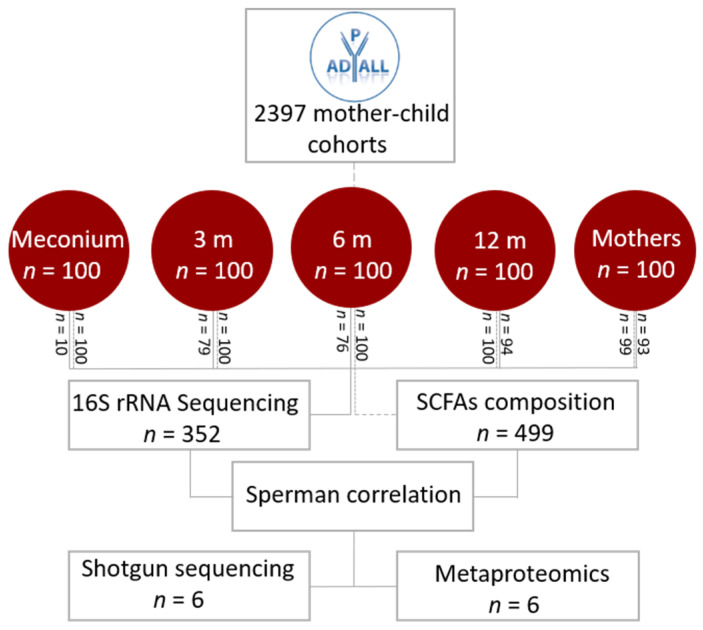
Flowchart of the study. The figure represents the workflow of the project. Sampling was performed by the Oslo University Hospital/University of Oslo, Østfold Hospital Trust and Karolinska Institutet, Stockholm [16]. In this study, we analyzed fecal samples from children in four different age groups (newborn, 3 months, 6 months, and 12 months) and their respective mothers, using a multiomics approach, which included 16S rRNA sequencing, short-chain fatty acids, shotgun sequencing, and metaproteomics. The stippled line represents the number (*n*) of fecal samples from infants analyzed at the different age groups at gas chromatography for short-chain fatty acid composition, and the full line represents the number of fecal samples from each age group that were analyzed by 16S rRNA after rarefaction and filtering for poor quality sequences.

Stool samples were diluted 1:10 in stool DNA stabilizer (PSP Spin Stool DNA Plus Kit, Invitek Molecular, Berlin, Germany) and stored at −80 °C prior to analysis. Gas chromatography was used to determine SCFA composition. Taxonomic composition was derived by amplification of the V3 to V4 region of 16S rRNA by PRK341F and PRK806R primers [17]. The amplicons were indexed using a combination of 16 forward and 30 reverse PRK modified primers with illumina indexes. The pooled library was thereafter sequenced on the Illumina MiSeq platform using Illumina’s MiSeq Reagent Kit v3 (Illumina Inc, San Diego, CA, USA). We used ASCA-ANOVA to determine if delivery mode, gender, age, and breastfeeding from 3 to 12 months were factors associated with the microbiota composition. The relationship between gut bacteria and SCFAs was investigated using Spearman correlation, and all shown correlations have a false discovery rate (FDR) corrected p-value of less than 0.05. Intracellular proteins from bacteria were extracted and analyzed in technical duplicates from six children at 12 months of age. A metagenome sequencing was performed to detect bacteria present in the stool samples and to reconstruct their genomes, unveiling their genomic information. This genomic information was further used as a database for metaproteomics data in order to observe protein expression related to gut function. The workflow of the study is illustrated in Figure 1, and detailed methods are available in supplementary materials and methods.

Sequencing data is available in the NCBI SRA database with identifier PRJNA609319. Proteome data has been uploaded via ProteomeXchange with identified PXD017844. Shotgun data are available upon request in TSD UiO (University of Oslo).

## 3. Results

### 3.1. Taxonomic Composition

Quality filtering and cut-off from raw illumina sequencing reads resulted in 352 samples with sufficient quality for downstream analysis. The cut-off was set to 5000 sequences per sample, as the observed species rarefaction curve showed saturation at approximately 5000 sequences for all infant age groups (Supplementary Figure S1).

The average number of unique species increased with age; 26 species were detected in meconium (first stool after birth), 37 at 3 months, 46 at 6 months, 72 at 12 months, and 183 in the mothers. The same pattern, characterized by the increased α-diversity with infant age, was also shown by Shannon–Wiener and Simpson’s index. Here, the mothers’ gut microbiota displayed the highest α-diversity index (Supplementary Figure S2). β-diversity showed a distinct clustering of the gut microbiota by the different age groups (Supplementary Figure S3).

**Figure 2:**
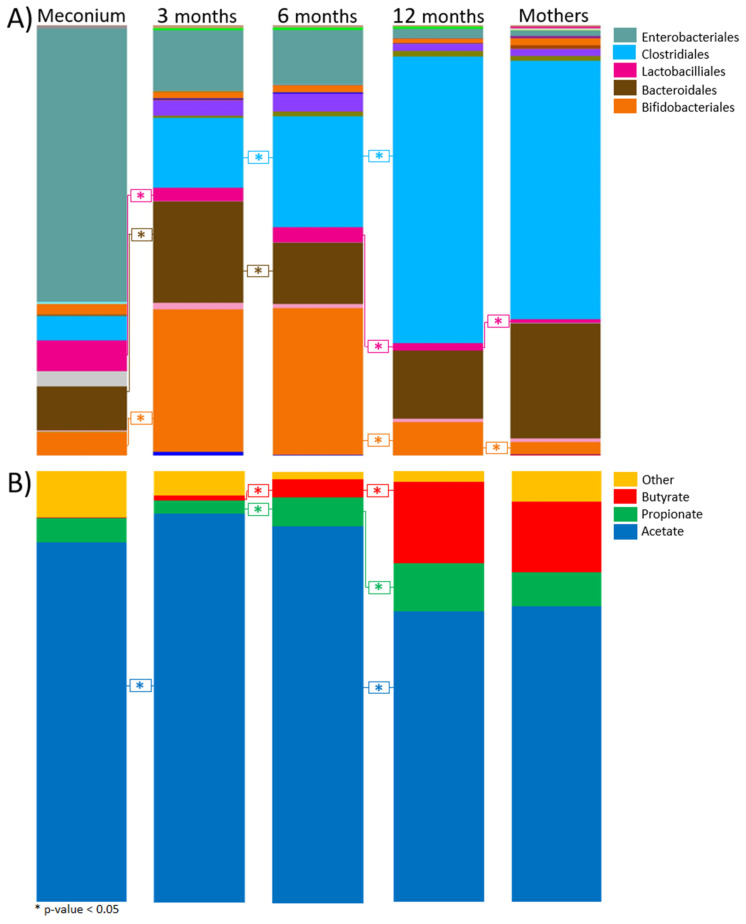
Taxonomic and short-chain fatty acid (SCFA) composition. The bar chart shows the relative abundance (%) of bacterial orders acquired from sequencing processed by the QIIME pipeline (**A**) and SCFAs composition (**B**) for the respective age groups. The dominant orders of bacteria and SCFAs in their respective colors are displayed on the top right. The asterisks (*) represent a *p*-value < 0.05, determined by Kruskal–Wallis–Dunn’s test, FDR correct by the Benjamini–Hochberg method. The exact p-values are shown Supplementary Tables S1 and S2. SCFAs illustrated represent percent based on average total SCFAs detected. SCFAs included in the “other” group are isobutyrate, isovalerate, and valerate.

**Figure 3:**
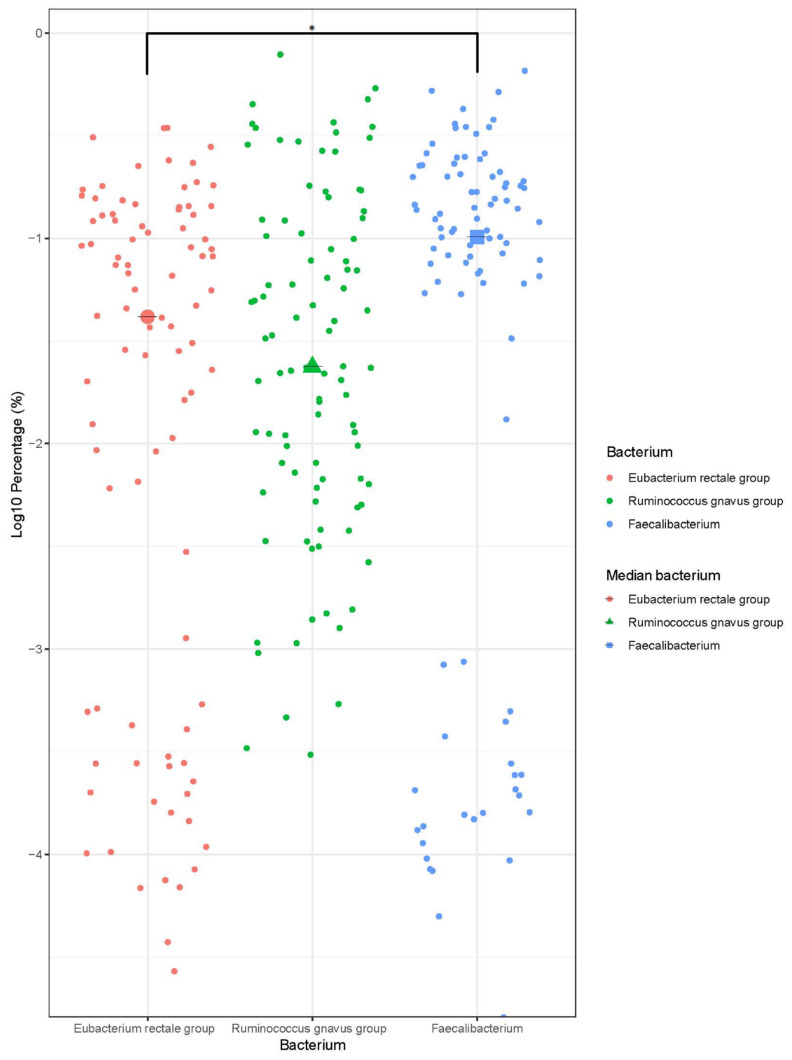
Relative abundance of *E. rectale*, *R. gnavus*, and *Faecalibacterium*, at 12 months. The dot plot shows the relative abundance (Log10 of percentage abundance) and median of the *E. rectale* group, *R. gnavus* group, and *Faecalibacterium*, at 12 months of age for all infants. The asterisk (*) represents a significant difference in relative abundance (*p* < 0.05, Wilcoxon rank sum test, FDR correct by Benjamini–Hochberg method) between the bacteria.

The infant gut microbiota showed the highest diversity at 12 months of age with the highest number of unique species, mostly derived from *Clostridiales* (Figure 2). *Clostridiales* increased significantly (*p* < 0.05, Kruskal–Wallis–Dunn’s test, FDR corrected by the Benjamini–Hochberg method) between the ages of 6 and 12 months, and represented approximately 67% of the gut bacteria at 12 months. The prominent genera/families within the *Clostridiales* were *Faecalibacterium* (13.1%), *Ruminococcus gnavus* group (9.4%), and *Eubacterium rectale* group (7.3%) (Figure 3). *Faecalibacterium* was significantly higher than the *E. rectale* group (*p* < 0.05, Wilcoxon rank sum, FDR by the Benjamini–Hochberg method).

### 3.2. Maternal and Infant Factors Association with Microbiota

**Figure 4:**
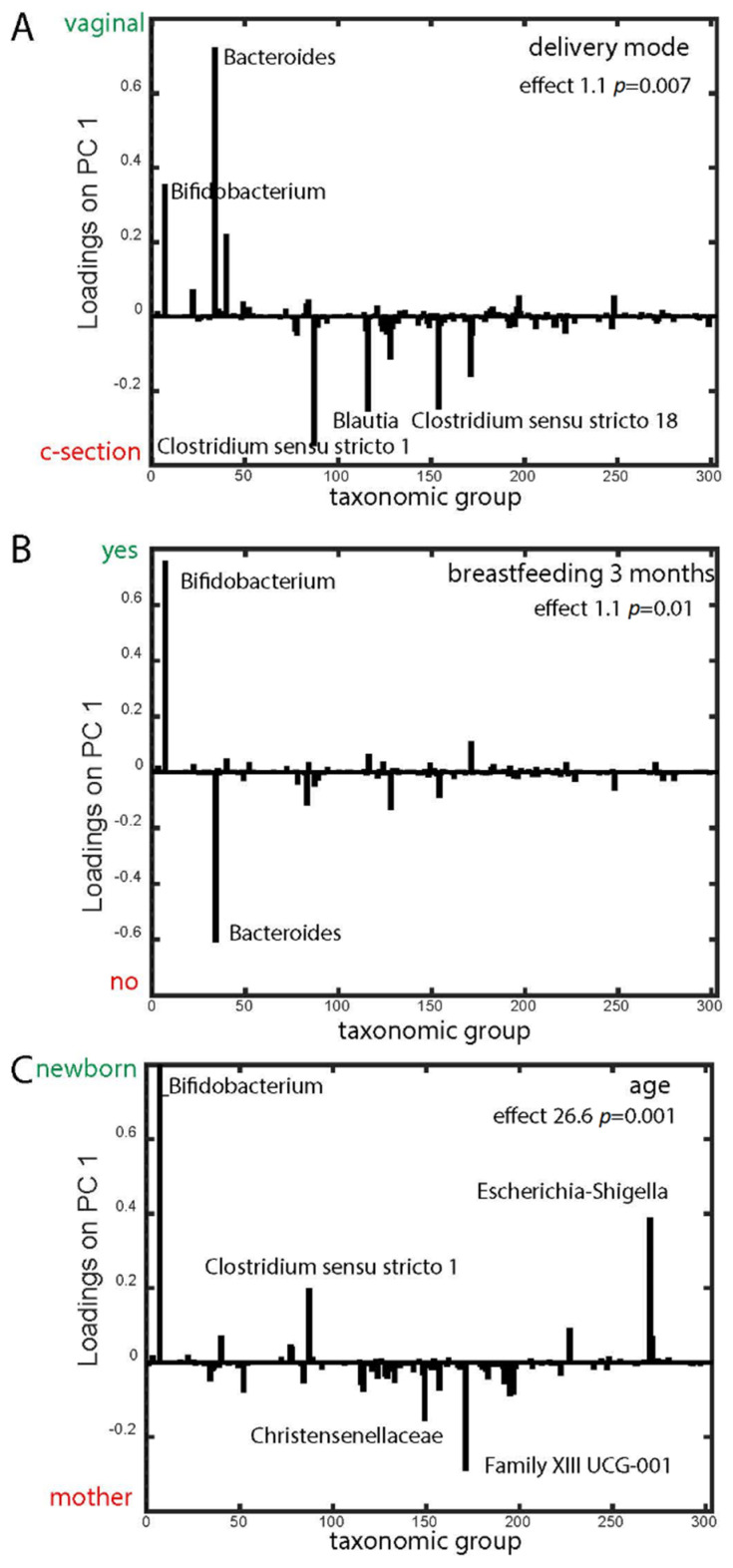
Metadata association with microbiota. ANOVA-simultaneous component analysis (ASCA-ANOVA) was used to determine the association of microbiota to known factors of the children. The Principal Component Analysis (PCA) plot shows the effect (Y-axis) of delivery method: vaginal and C-section (**A**), breastfeeding between 3 and 6 months of age (**B**), and age (**C**) based on the taxonomic groups acquired from 16S rRNA sequencing (X-axis). (**C**) The Y-axis is a gradient of age, where the effect points towards a young age or old age, showing the outer points of the scale: newborns and mother.

Age, delivery mode, and breastfeeding at 3 months of age showed significant associations with microbiota, with increasing age explaining the majority of the variance (Table 1). Vaginal delivery was associated with *Bifidobacterium* and *Bacteroides*, while *Clostridia* were associated with caesarean section (Figure 4A). At 3 months, breastfeeding was associated with *Bifidobacterium,* while no breastfeeding was associated with *Bacteroides* (Figure 4B). *Bifidobacterium* and *Escherichia/Shigella* were associated with young age, while *Clostridia* were associated with infants at 12 months and mothers (Figure 4C).

### 3.3. SCFAs Composition

Acetate was the dominant SCFA in mothers and in all infant age groups. The highest relative abundance of acetate in infants was observed at 3 months of age (90.1 ± 7.9%), while the lowest observed was at 12 months (67.4 ± 5.1%) (Figure 2). Propionate was present in all age groups and increased significantly between 3 and 12 months (Supplementary Table S2), with an overall ratio of 11.2 ± 1.3% at 12 months of age. Similar to propionate, butyrate increased significantly between 3 and 12 months of age. We detected a four-fold increase of the relative abundance of butyrate between 6 and 12 months of age (*p* < 0.05, Kruskall–Wallis–Dunn’s test, FDR corrected with Benjamini–Hochberg), with butyrate representing 18.9 ± 2% of the total SCFAs at 12 months. In general, the absolute values of SCFA in relation to 16S rRNA gene copy number decreased with increasing age (Supplementary Figure S4A).

### 3.4. Bacterial and SCFA Correlation

**Figure 5:**
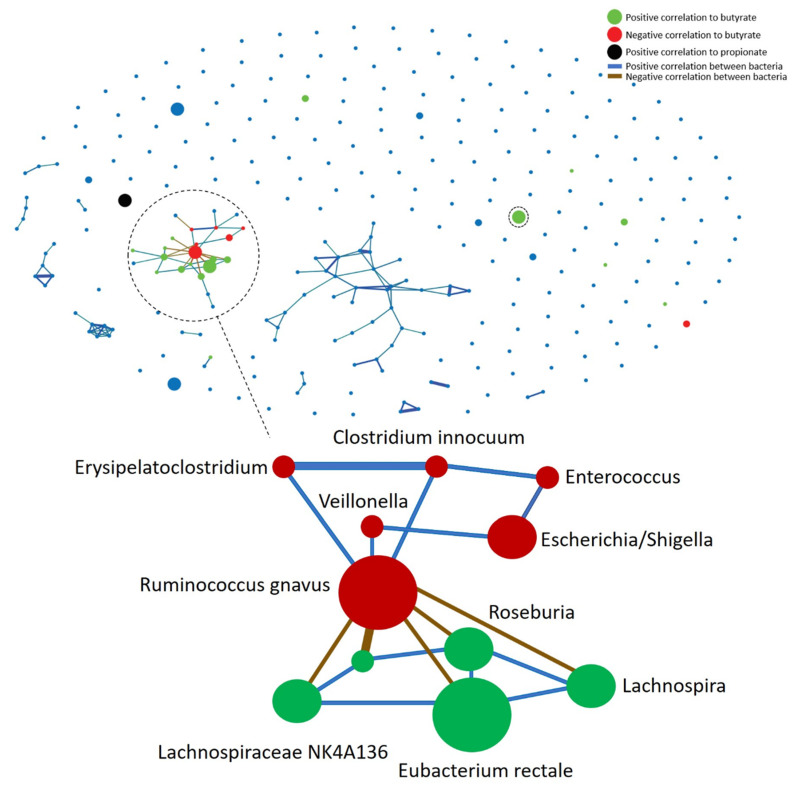
Bacterial and SCFA correlations at 12 months. The illustration shows all OTUs from 16S rRNA represented as nodes, with color indicating their correlation to SCFAs; blue = no correlation, red = negative correlation to butyrate, green = positive correlation to butyrate, and black = positive correlation to propionate. The three different node sizes represent the general abundance of the respective bacteria. The thickness of the lines between nodes represents a correlation between the bacteria, of which a thick line is a strong correlation. Blue lines indicate a positive correlation between the bacteria, while brown lines indicate a negative correlation. Prominent nodes in the networks are highlighted with their respective OTU taxonomy. The highlighted green circle with positive association to butyrate but without correlations to other bacteria was assigned to *Faecalibacterium*.

At 3 months of age, we observed *Anaeroglobus, Anaerotruncus, Ruminococcus torques¸* and *Eubacterium xylanophilum* to have a positive correlation to the relative amount of butyrate (Supplementary Figure S5A). However, at 6 months of age, we observed a positive correlation between *Sphingomonas* and the relative amount of propionate, while *Stenotrophomonas* had a positive correlation to the relative amount of both acetate and propionate (Supplementary Figure S5B). *Haemophilus* in mothers were positively correlated to the relative amount of butyrate, while *Akkermansia* were negatively correlated (Supplementary Figure S5C). At 12 months of infant age, we observed a correlation pattern revealing two distinct bacterial networks, one characterized by *Eubacterium rectale’s* positive correlation to the relative amount of butyrate and the other defined by *Ruminococcus gnavus’* negative correlation to the relative amount of butyrate (Figure 5).

*E. rectale* represented the most abundant species in the network, correlating positively with both the relative amount of butyrate and the bacteria *Roseburia, Lachnospiraceaea* NK4A136, and *Lachnospira*. Bacteria in the *E. rectale* network were all negatively correlated to *R. gnavus*. *R. gnavus* showed a negative correlation to the relative amount of butyrate and a positive correlation to *Erysipelatoclostridium*, *Veillonella*, and *Clostridium innocuum*, with *Enterococcus* and *Escherichia/Shigella* also showing a positive correlation to members of the *R. gnavus* network. All bacteria related to the *R. gnavus* network had a negative correlation to butyrate (Figure 5). Infants with the *E. rectale* network had significantly higher absolute abundance of butyrate per 16S gene copy (*p* < 0.05, Mann–Whitney–Wilcoxon test) than infants with the *R. gnavus* network at 12 months of age (Supplementary Figure S4B).

Infants were divided into groups based on the cumulative sum of the relative abundance of bacteria with a positive or negative correlation to butyrate, and a positive correlation between the bacteria. This resulted in 43 infants being categorized with the *E. rectale* network, 27 with the *R. gnavus* network, 19 with none, and 5 with both at 12 months of infant age (*p* < 0.05, chi-square test).

The presence of *R. gnavus* in infants with a high relative abundance of the *R. gnavus* network at 12 months of age was not significantly associated with the presence of *R. gnavus* at 6 months of age (*p* > 0.05, chi-square test). However, the presence of *R. gnavus* in infants at 6 months of age was significantly associated with the *R. gnavus* presence at 3 months of age (*p* < 0.05, chi-square test). The eight infants with a high relative abundance of *R. gnavus* at 3 months had a high relative abundance of the *E. rectale* network at 12 months of age, with one of the infants still having a high relative abundance of the *R. gnavus* network (>10% of the microbiota) as well.

The presence of the *E. rectale-* and *R. gnavus* networks at 12 months of infant age did not significantly correlate to delivery mode, gender, breastmilk feeding, or introduction to solid foods (*p* > 0.05, chi-square test) (Supplementary Table S3).

### 3.5. Metaproteome Analysis of E. Rectale and R. Gnavus Network Associated Bacteria

**Figure 6:**
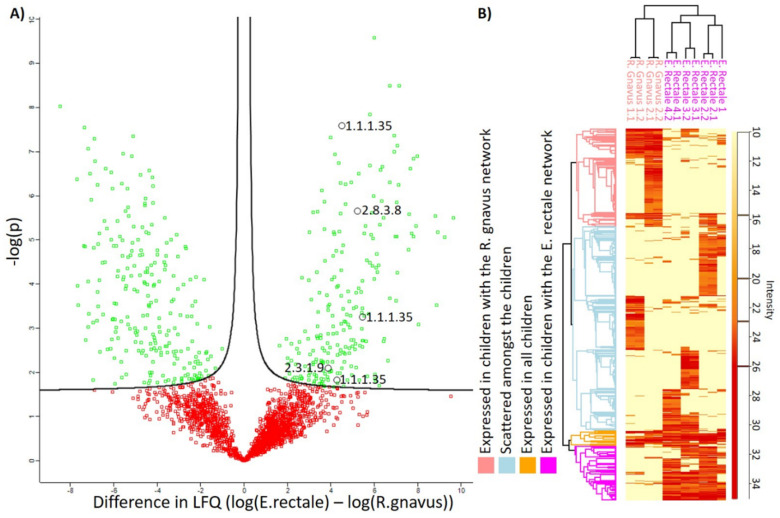
Protein expression. (**A**) shows a Volcano-plot highlighting (green) proteins differentially expressed between children with *E. rectale* and the *R. gnavus* community. The volcano plot was created using the Perseus software. Proteins marked as black circles represent significant differential expression in proteins related to the butyrate pathway with their respective Enzyme Commission (E.C.) numbers. (**B**) shows the Log2 Label-Free Quantification (LFQ) intensity of the proteins, with missing values imputed with the constant 10. The intensity is represented as a gradient from light yellow (not detected) to red (highly abundant). Clustering is shown on the left, including four clusters, expressed in children with the *R. gnavus* network, *E. rectale* network, scattered, or expressed in all. A dendrogram is included at the top, to show the similarities and dissimilarities between the technical replicates and the children with *E. rectale*- or *R. gnavus*-dominated communities, with their respective technical duplicate (e.g., “*R. gnavus* 1.2” annotates child number one with the *R. gnavus* network and technical duplicate two). *E. rectale* infant 1 did not have any technical duplicates.

Intracellular bacterial proteins were extracted and analyzed in technical duplicates from six infants, excluding one, based on their relative abundance of *E. rectale*, *R. gnavus*, and butyrate at 12 months of infant age. Of the 2212 detected protein IDs, 511 were observed to be significantly different in abundance between infants having a high relative abundance of either *E. rectale* or *R. gnavus* (Figure 6) (*p* < 0.05, t-test with permutation-based FDR and with missing values imputed). In addition, 253 protein IDs were only detected in infants with the *E. rectale* network and 80 detected only in infants with the *R. gnavus* network.

**Figure 7:**
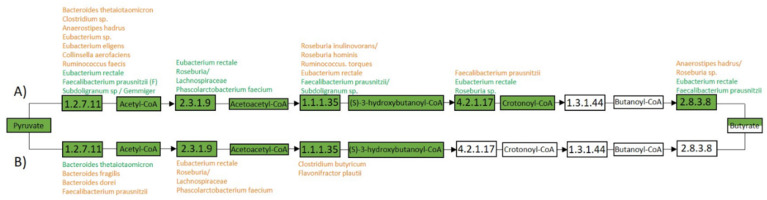
Protein presence related to butyrate production. The figure gives an overview of bacterial proteins (E.C. number) in relation to the butyrate pathway butyryl-CoA:Acetate CoA-transferase. The figure shows proteins detected (green box) in infants with the *E. rectale* network (**A**), or *R. gnavus* network (**B**). Bacterial taxonomy is shown next to each E.C. number, representing the bacterial source of the given protein. The bacterial sources are divided by two different colors, where orange represents detection in three or fewer samples in (**A**) or two or fewer in (**B**), and green represents detection in four or more in (**A**) or three or more in (**B**).

We detected the enzymes needed to convert acetyl-CoA to butyrate via the butyryl-CoA:Acetate CoA-transferase pathway, with several of the enzymes being mapped to the butyrate-associated bacteria *E. rectale*, *F. prausnitzii*, and *Roseburia* based on their genomic information. Enoyl-CoA, 3-hydroxybutyryl-CoA dehydratase, and butyryl-CoA:Acetate CoA-transferase were only detected in fecal samples of infants with the *E. rectale* network (Figure 7). We did not detect enzymes related to propionate production to be mapped to the propionate associated bacteria, *Bacteroides*, apart from an enzyme needed for acetyl-CoA to malonyl-CoA conversion (Supplementary Figure S6). However, we detected several enzymes in relation to propionate production that were mapped to *Eubacterium halli*, *Lachnospiraceae,* and *F. prausnitzii*. Conversion from propanoyl-phosphate to propionate was only detected and mapped to *Bifidobacteria* (*breve, pseudocatenulatum, longum,* and *bifidum*). As for enzymes related to acetate production, we detected *F. prausnitzii, E. halli, Blautia, Lachnospiraceae, B. breve, B. pseudocatenulatum, B. longum,* and *B. bifidum* to be potential acetate producers.

**Figure 8:**
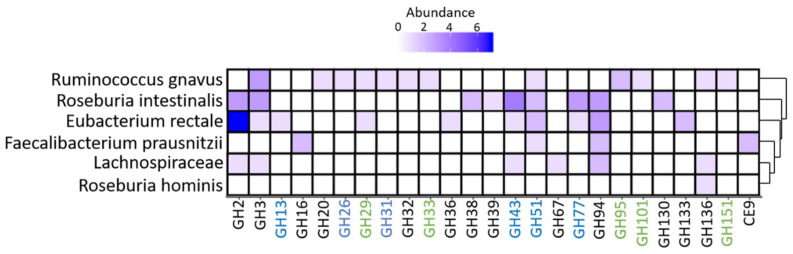
Expressed glycoside hydrolases/carbohydrate esterases. The figure shows proteins expressed within the glycoside hydrolase and carbohydrate esterase groups expressed based on the contiguous sequences assembled from shotgun sequencing and protein expression derived from nanoLC-Orbitrap tandem mass spectrometry (MS/MS). The abundance represents the number of unique proteins expressed from a given taxa within the glycoside hydrolases (GH) or carbohydrate esterase (CE) group. The y-axis shows the relevant taxonomies, and the dendrogram represents the Euclidian distance between the taxonomic groups based on GH and CE expression. The plot was created using ggplot2 [18]. GH numbers related to mucus and fucose degradation are marked in green, and degradation of starch, glycogen, and hemicellulose are marked in blue.

Of the proteins detected, we observed glycoside hydrolases (GHs), which potentially degraded a variety of dietary fibers, including hemicellulose (GH43 and GH51), starch/glycogen (GH13 and GH77), cellobiose/chitobiose (GH94), and fucose (GH29), in addition to broad specific glucosidases (GH2 and GH3), which were potentially expressed by *E. rectale* (Figure 8). GHs mapped to *R. gnavus* consisted of GHs related to mucin (GH33 and GH101), fucose (GH29, GH95, and GH151), human milk oligosaccharides (GH20), mannose (GH26), starch (GH31), sucrose (GH32), and a broad-specific glucosidase (GH3).

## 4. Discussion

The highest relative abundance of butyrate was observed at 12 months of infant age with the presence of the *E. rectale* network and *F. prausnitzii*, suggesting an additive effect in butyrate production. Based on our results, we believe that the *E. rectale* network co-occurring with *F. prausnitzii* could play a key role in the elevated relative abundance of butyrate. Earlier studies have observed that both *F. prausnitzii* and *E. rectale* are important butyrate producers in the adult gut [19], which suggests that the transition from 6 to 12 months of infant age may represent a crucial transition stage with respect to establishment of butyrate producers. We only detected one enzyme in relation to propionate production by *Bacteroides*, which may reflect a lower resolution of detected enzymes from other pathways due to selecting samples with a high relative abundance of butyrate, *E. rectale,* and *R. gnavus*.

Infants with a low relative abundance of butyrate at 12 months had a high relative abundance of the *Ruminococcus gnavus* network. *R. gnavus* can potentially degrade protein-linked human milk oligosaccharides (HMOs) [20], in addition to utilizing mucin glycans from the host [21]. Their potential ability to degrade HMOs and host glycans might explain their early presence in the gut. In addition, *R. gnavus* has been observed to be overrepresented in infants with a higher risk of developing allergic disease, including allergic rhinitis, asthma, and atopic dermatitis [22]. The potential pathogenicity of *R. gnavus* is suggested to be related to mucin degradation [23], by trans-sialidase or fucosidase, depending on the strain [21,24]. Concurrently, we observed glycoside hydrolases related to mucus- and HMO degradation to potentially be expressed by *R. gnavus*. As only 8.4% of the PreventADALL infants had documented atopic dermatitis at 12 months of age [25], we did not investigate microbiota-atopy associations, as the current study is underpowered to address these questions. We cannot exclude expression of other GHs, as the method used in the current study is only able to analyze intracellular proteins. In addition, we could not identify polysaccharide utilization loci (PUL), nor complete phosphotransferase system (PTS); therefore, the expressed proteins might not necessarily reflect the bacteria’s metabolism.

The infants in our study had lower relative abundance of butyrate at 6 months (4.1%) than Estonian (12.3%) and Swedish (7.8%) infants [26], but had the largest increase in butyrate ratio between 6 and 12 months, while Estonian infants had the lowest. The earlier presence and slower emergence of butyrate in Estonian infants could potentially be linked to an earlier introduction of fibers, which may result in lower allergy occurrence [27], but this needs to be explored further.

The bacterial successional patterns in infants in the present study were similar to previous observations in other studies, with *Bifidobacterium* dominating the gut up to 6 months of age and then shifting to Firmicutes dominance at 12 months [28,29]. *E. rectale* has previously been observed already from 6 months of age, but increases its prevalence and abundance at 12 months of infant age [30], which coincides with our results. *R. gnavus* has been observed to be prevalent already at 1 month of age [31]. This early presence might be a result of its potential to degrade HMOs and host glycans. The abundance of *R. gnavus* has been shown to increase with age, particularly in allergic infants [22]. We observed an increase in *R. gnavus* prevalence with age; however, we did not find its presence at 12 months of age to be dependent on its presence at 3 months of age.

The association of *Bacteroides* with vaginal delivery was recently observed in both The Environmental Determinants of Diabetes in the Young (TEDDY) and the Baby Biome studies (BBS) [8,32], and association between *Bifidobacteria* and vaginal delivery has also been observed earlier [33]. Cesarean-section is often associated with a higher abundance of Firmicutes, which correspond with our observation of *Clostridiales* being associated with cesarean-section [34]. The observed negative association of *Bacteroides* with breastfeeding at 3 months of age has not been described previously and could possibly be explained by the fact that our study population was sampled from a general population, unlike most other large-scale studies. The maternal and infant factors assessed in this study did not explain why some infants had either the *E. rectale-* or the *R. gnavus* network at 1 year. Breastmilk feeding and introduction to solid foods are categorical variables, and do not reflect the intake amount or other foods introduced. The breastfeeding variable does not represent exclusive breastfeeding for the given time frame. We therefore cannot rule out that infant diet might have played a role in the presence of the networks.

## 5. Conclusions

In conclusion, we observed a four-fold increase in the relative abundance of butyrate from 6 to 12 months of infant age, with the *Eubacterium rectale-* and *Ruminococcus gnavus* networks being positively and negatively correlated to butyrate, respectively. Furthermore, we detected expressed enzymes from the butyryl-CoA:Acetate CoA-transferase pathway that might originate from *E. rectale* and *Faecalibacterium prausnitzii*. In addition, glycoside hydrolases related to dietary fiber degradation were potentially expressed by *E. rectale*. We believe these results suggest that the transition from 6 to 12 months of infant age represents a crucial transition stage with respect to establishment of key butyrate-producing bacteria.

## Figures and Tables

**Table 1:** Maternal and infant factors associations to operational taxonomic units (OTUs). The table shows the effect (%) and p-value for delivery mode, gender, and breastfeeding, between 3 and 6 months, 6 and 9 months, and 9 and 12 months, and infant age from 0–12 months.

Term	Effect (%)	*P*-Value
Delivery mode	1.06	0.01
Infant gender	0.26	0.25
Breastfeeding 3–6 months	1.12	0.01
Breastfeeding 6–9 months	0.60	0.73
Breastfeeding 9–12 months	0.72	0.05
Infant age	26.63	0.001

## Supplementary Materials

The following are available online at https://www.mdpi.com/2073-4425/11/11/1245/s1. Supplementary 1: Materials and Methods. Figure S1: Rarefaction curve of observed species, Figure S2: α diversity, Figure S3: β-Diversity, Figure S4: Total short-chain fatty acids per 16S rRNA gene copy, Figure S5: Bacterial correlation to SCFA in all age groups, Figure S6: Expressed enzymes related to the propionate production pathway. Table S1: *p*-values Kruskall-Wallis-Dunn’s test for bacterial orders, Table S2: *p*-values Kruskall-Wallis Dunn’s test for SCFAs, Table S3: Maternal and infant factors associated with the presence of either *E. rectale* or the *R. gnavus* network in infants.
