# Supplementary material for: Butyrate Levels in the Transition from an Infant- to an Adult-Like Gut Microbiota Correlate with Bacterial Networks Associated with Eubacterium Rectale and Ruminococcus Gnavus
Source: Genes (Basel). 2020 Oct 22;11(11):1245. doi: 10.3390/genes11111245 (PMC7690385; doi:10.3390/genes11111245)
Supplement: Supplementary file 1 [file genes-11-01245-s001.pdf]

Materials and Methods Supplementary

Sample information

Fecal samples used in this study derived from the first 100 mother-child cohorts who attended at least three of the four follow-up investigations in the PreventADALL study. Infants were randomized to one of four groups by skin and food interventions[1]. From the 100 mother-child pairs, 99 pairs had available longitudinal sampling data from at least three of the four infant samples and from their respective mother approximately 18 weeks into pregnancy. Thirteen samples were mislabeled, although within the correct age group, so these samples were included in the 16S rRNA and SCFAs analysis as these were not analyzed longitudinally within each mother-child pair, but as a population of age groups over time.

Sample preparation

All fecal samples were diluted 1:10 in stool DNA stabilizer (PSP Spin Stool DNA Plus Kit, Invitek Molecular, Berlin, Germany) and stored at -80°C prior to analysis. Fecal samples were homogenized and pulse centrifuged at 1200 rpm for 8 seconds for easier extraction. From the 1:10 diluted samples, 300µl and 100µl aliquots were used for 16S rRNA sequencing and SCFA composition, respectively.

Short-chain fatty acid analysis

The aliquots were diluted 1:1 with MilliQ-water, and then 1:1 with an internal standard, containing 2% formic acid with 500µM 2-methylvaleric acid. Samples were centrifuged at 13 000 rpm for 10 minutes. The supernatant was filtered with 0.2µM filter columns (VWR, USA) at 10 000 rpm for 5 minutes. The eluate was transferred to gas chromatograph (GC) vials (VWR, USA) and applied to the gas chromatograph (Trace 1310 equipped with an autosampler, ThermoFisher Scientific) with ramping temperatures from 90°C to 150°C for 6 minutes and 150°C to 245°C for 1.9 minutes. 0.2µl was applied with a split injection to a Topaz 4.0mm drilled uniliner (Restek), using helium as the carrier gas with 2.5ml/min column flow, 3 ml/min purge flow and 200 ml/min split flow. The column used was a Stabilwax DA 30m, 0.25mm ID, 0.25µM (Restek), with a flame ionization detector analyzing the analytes. The chromatograms were processed with the Chromeleon 7 software.

A standard with 300µM acetic acid, 12µM propionic acid, 8µM isobutyric acid, 12µM butyric acid, 8µM isovaleric acid, 8µM valeric acid, 25µM internal standard and 1% formic acid was applied twice in between every 10th sample to detect shifts or variabilities. All acids used were purchased from Sigma-Aldrich, Germany.

16.S rRNA sequencing

Bacterial cells in fecal sample aliquots were disrupted using 0.2g <106µm acid-washed glass beads (Sigma-Aldrich, Germany), 0.2g 425-600µm acid-washed glass beads (Sigma-Aldrich, Germany) and 2×2.5-3.5mm acid-washed glass beads before being processed twice on a FastPrep 96 (MP Biomedicals, USA) at 1800rpm for 40 seconds. The samples were centrifuged at 13 000 rpm for 5 minutes before DNA was extracted using LGC Mag Midi Nucleic acid extraction kit (LGC genomics, UK). The V3 to V4 region of 16S rRNA was amplified using PRK341F and PRK806R primers[2] at 95°C for 15 minutes followed by 25 cycles of 95°C for 30 seconds, 55°C for 30 seconds, and 72°C for 45 seconds, before a final step at 72°C for 7 minutes. Cycles were increased to 30 for meconium. Reactions contained 2µl DNA template with 1× HotFirePol Blend Master Mix Ready to Load (Solis BioDyne, Germany) and 0.2µm PRK forward and reverse primers. Samples were purified using 1× Sera Mag beads to the DNA volume, following AMPure's protocol on a Biomek 3000 (Beckman Coulter, USA).

Index PCR was performed with a combination of 16 forward and 30 reverse modified PRK primers with Illumina indexes. Samples were amplified at 95°C for 5 minutes followed by 10 cycles of 95°C for 30 seconds, 55°C for 60 seconds, and 72°C for 45 seconds, before a final step of 72°C for 7

minutes. Each reaction consisted of 1× FirePol Master Mix Ready to Load (Solis BioDyne, Germany), 0.2µM forward & reverse primers, nuclease free-water (VWR, USA) and 1µl DNA. The DNA concentration was quantified following Qubit's protocol, normalized and pooled on a Biomek 3000. The pooled sample was split in two for quantification and sequencing. Samples for quantification were first subjected to droplet generation using BioRad QX200™ – Droplet Generator, before being amplified at 95°C for 5 minutes followed by 40 cycles of 95°C for 30 seconds, 60°C for 30 seconds, and 72°C for 45 seconds before the last two steps at 4°C for 5 minutes and 90°C for 5 minutes before quantification on BioRad QX200 – Droplet Reader. The reactions contained 1× Super mix for EvaGreen (BioRad, USA), 0.2µM Illumina colony forward & reverse primer, 2.4µl DNA template and PCR water. The second part of the sample was diluted to 6 pM DNA with 15% PhiX following Illumina's instructions, with the exception of using nuclease-free water instead of Tris and sequenced on Illumina MiSeq.

Quantitative Insights Into Microbial Ecology (QIIME)

The 16S rRNA data were analyzed with Quantitative Insights Into Microbial Ecology (QIIME) pipeline[3]. QIIME v.1.9.1 was used to assemble forward and reverse reads and split them into their respective samples. Usearch v8 was used to check reads for chimeras, and OTUs with a 97% or higher 16S rRNA identity were created and assigned taxonomy by the SILVA 128 database[4]. Two sequencing runs were performed resulting in 30 878 312 ssDNA fragments. The cut-off was set at 5 000 dsDNA fragments, resulting in 352 samples with sufficient depth and quality. This was distributed as follows: meconium n=10, 3 months n=79, 6 months n=76, 12 months n=94 and mother n=93.

Shotgun metagenome sequencing

Six samples were selected for Shotgun sequencing, based on the presence and absence of *E. rectale, R. gnavus* and butyrate. The samples were processed using the Nextera XT DNA Library Preparation Kit (Illumina Inc, San Diego, CA, USA), following the manufacturer's instructions and sequenced on the Illumina MiSeq platform twice, resulting in approximately 4.7 Gb.

Processing shotgun data

Human DNA was removed using bowtie2 v2.3.5.1 [5] and samtools v1.9 [6], and reads were trimmed using trimmomatic v0.36 [7], with the following parameters: Leading: 10, Trailing: 10, Slidingwindow: 5:20 and minlen: 32. Assembly was performed using MetaSPADES v3.13.1 [8], and the contigs created qualitatively assessed using MetaQuast v5.0.2[9]. Prodigal v2.6.3 [10] was used for gene prediction, whereas the InterProScan v5.39-77.0 [11] consortium predicted proteins and pathways. The predicted proteins from shotgun sequencing were further used as a sample-specific protein sequence database for the metaproteomic analysis. Contig taxonomy was annotated by a customized Kraken2 database [12], which involved the standard database with inclusion of *Ruminococcus gnavus* ATCC 29147, *Bacteroides uniformis* AF14-42, *Eubacterium rectale* AF36-2BH retrieved from NCBI.

Protein extraction and quantification

Intracellular proteins were extracted from the gut bacteria derived from the same six children used for shotgun sequencing. All samples were run in technical duplicates, excluding one due to an insufficient amount of fecal material.

An indirect double filtering process was performed on 0.2g fecal material. Fecal samples were suspended in 10mL TBS (Tris-based saline buffer), before being filtered through a 20µm filter (Merck™ Nylon-Net Steriflip™ Vacuum Filter Unit, Fisher Scientific), homogenized at 30 000 rpm for 60s (VDI12, VWR), centrifuged at 4000g for 10 minutes and thereafter resuspended in 10mL TBS and filtered through a 0.22µm nitrocellulose filter [13]. The filter was cut into smaller pieces, mixed with lysis buffer, and bacteria were mechanically lysed as explained in the 16S rRNA sequencing section.

The lysis buffer contained 50mM Tris-HCl, 200mM NaCl, 0.1% Triton-X100, 10mM Dithiothreitol (DTT) and 4% Sodium Dodecyl Sulfate (SDS). Protein quantification was performed using the BCA-DC kit, following the manufacturer's protocol, before applying 40µg protein to an SDS-gel. The gel was run for approximately 2 minutes. The proteins were fixed using 50% methanol and 10% glacial acetic acid for 1 hour with gentle agitation, and thereafter stained using 0.1% Coomassie Brilliant Blue R-250 with 50% methanol and 10% glacial acetic acid, and destained with 40% methanol and 10% glacial acetic acid.

In-gel reduction, alkylation and digestion

The gel band containing proteins was cut into approximately 1×1mm pieces and de-colored with Milli-Q water (MQ) and incubated at 15 minutes in room temperature (RT). MQ was removed and 50% acetonitrile (ACN) with 25 mM ammonium bicarbonate was added and incubated 15 minutes at RT. 100% ACN was added and incubated for 5 minutes at RT, before it was removed and then air-dried. Reduction and alkylation were performed by adding 10mM DTT with 100 mM ammonium bicarbonate for 30 minutes at 56ºC. The samples were cooled before the solution was removed and 55mM iodoacetamine (IAA) with 100mM ammonium bicarbonate was added and incubated in the dark for 30 minutes in RT. The IAA solution was removed, and 100% ACN was added and incubated for 5 minutes in RT before the solution was removed and air-dried for 2 minutes. For digestion 40ng of Trypsin solution was added and gel-pieces were incubated overnight at 37ºC, thereafter 1% trifluoroacetic acid (TFA) was added and the gel-pieces were sonicated in a water bath for 15 minutes. The resulting peptides were desalted using C_{18} solid phase ZipTips according to the manufacturer's instructions.

nanoLC-Orbitrap MS/MS

Dried peptides were dissolved in a solution (0.1% TFA and 2% ACN in water) and analyzed on a nanoLC-MS/MS system (Dionex Ultimate 3000 UHPLC; Thermo Scientific, Bremen, Germany) connected to a Q-Exactive mass spectrometer (Thermo Scientific, Bremen, Germany). In brief, peptides were loaded onto a trap column (Acclaim PepMap100, C18, 5 µm, 100 Å, 300 µm i.d. × 5 mm) and then backflushed onto a 50 cm × 70 µm analytical column (Acclaim PepMap RSLC C18, 2 µm, 100 Å, 75 µm i.d. × 50 cm, nanoViper). A 120 min gradient from 3.2 to 36% solution B (99.9 % ACN, 0.1% formic acid) was used for separation of the proteins, at a flow rate of 300 nl/min. The Q-Exactive mass spectrometer was set up as follows (Top5 method): a full scan (300-1600 m/z) at R=70.000 was followed by (up to) 12 MS2 scans at R=17500, using an NCE setting of 28. Singly charged precursors were excluded for MSMS, as were precursors with z>5. Dynamic exclusion was set to 20 seconds.

The MS raw files were analyzed, identified and quantified using MaxQuant version 1.6.6.0, with the MaxLFQ algorithm [14,15] implemented for label-free quantitative detection of proteins. In brief, the raw files were searched against the sample-specific protein sequence database and against the human genome (*Homo sapiens*, 73952 sequences). The sequences database was complemented with common contaminants, such as human keratin, trypsin and bovine serum albumin, as well as reversed sequences of all protein entries to estimate the false discovery rate. Oxidation of methionine's, protein N-terminal acetylation, deamination of asparagine and glutamine, and conversion of glutamine to pyro-glutamic acid were used as variable modifications, while carbamidomethylating of cysteine residues was used as a fixed modification. Two missed cleavages of trypsin were allowed, and all identifications were filtered in order to achieve a protein false discovery rate of 1% using the target-decoy strategy in Perseus version 1.6.6.0 [16], resulting in 2215 proteins. Functional annotation of the proteins was determined by a combination of the InterProScan database [11], Pfam database [17] and manual search using protein BLAST. Proteins were annotated as GH or CE from the DBCan meta server v8 [18] and all GH and CE annotated equally by at least two of the three databases were included (DIAMOND, HMMER, Hotpep).

Statistical analysis

Statistical analysis was performed in Rstudio [19] and MatLab [20]. Kruskall-Wallis-Dunns test was performed with R version 3.4.3 and R-package PMCMR plus (2018). Correlation of bacterial profiles to SCFAs was performed by using Spearman correlations, with a p-value less than 0.05, FDR corrected using the Benjamini-Hochberg method in the MatLab programming environment [20]. Correlation of the metadata to 16S using ASCA-ANOVA was performed in the MatLab environment, while metadata correlation to children with the bacterial networks was performed using a chi-square test.

Reference

1. Lødrup Carlsen, K.C., et al., *Preventing Atopic Dermatitis and ALLergies in Children—the PreventADALL study.* 2018. **73**(10): p. 2063-2070.
2. Yu, Y., et al., *Group-specific primer and probe sets to detect methanogenic communities using quantitative real-time polymerase chain reaction.* 2005. **89**(6): p. 670-679.
3. Caporaso, J.G., et al., *QIIME allows analysis of high-throughput community sequencing data.* Nature methods, 2010. **7**(5): p. 335-336.
4. Pruesse, E., et al., *SILVA: a comprehensive online resource for quality checked and aligned ribosomal RNA sequence data compatible with ARB.* Nucleic acids research, 2007. **35**(21): p. 7188-7196.
5. Langmead, B. and S.L. Salzberg, *Fast gapped-read alignment with Bowtie 2.* Nature Methods, 2012. **9**(4): p. 357-359.
6. Li, H., et al., *The Sequence Alignment/Map format and SAMtools.* Bioinformatics (Oxford, England), 2009. **25**(16): p. 2078-2079.
7. Bolger, A.M., M. Lohse, and B. Usadel, *Trimmomatic: a flexible trimmer for Illumina sequence data.* Bioinformatics (Oxford, England), 2014. **30**(15): p. 2114-2120.
8. Nurk, S., et al., *metaSPAdes: a new versatile metagenomic assembler.* Genome research, 2017. **27**(5): p. 824-834.
9. Mikheenko, A., V. Saveliev, and A. Gurevich, *MetaQUAST: evaluation of metagenome assemblies.* Bioinformatics, 2015. **32**(7): p. 1088-1090.
10. Hyatt, D., et al., *Prodigal: prokaryotic gene recognition and translation initiation site identification.* BMC bioinformatics, 2010. **11**: p. 119-119.
11. Jones, P., et al., *InterProScan 5: genome-scale protein function classification.* Bioinformatics (Oxford, England), 2014. **30**(9): p. 1236-1240.
12. Wood, D.E. and S.L. Salzberg, *Kraken: ultrafast metagenomic sequence classification using exact alignments.* Genome biology, 2014. **15**(3): p. R46-R46.
13. Xiong, W., et al., *Development of an enhanced metaproteomic approach for deepening the microbiome characterization of the human infant gut.* Journal of proteome research, 2015. **14**(1): p. 133-141.
14. Cox, J., et al., *Accurate proteome-wide label-free quantification by delayed normalization and maximal peptide ratio extraction, termed MaxLFQ.* Molecular & cellular proteomics : MCP, 2014. **13**(9): p. 2513-2526.
15. Cox, J. and M. Mann, *MaxQuant enables high peptide identification rates, individualized p.p.b.-range mass accuracies and proteome-wide protein quantification.* Nature Biotechnology, 2008. **26**(12): p. 1367-1372.
16. Tyanova, S. and J. Cox, *Perseus: A Bioinformatics Platform for Integrative Analysis of Proteomics Data in Cancer Research*, in *Cancer Systems Biology: Methods and Protocols*, L. von Stechow, Editor. 2018, Springer New York: New York, NY. p. 133-148.
17. El-Gebali, S., et al., *The Pfam protein families database in 2019.* Nucleic Acids Research, 2018. **47**(D1): p. D427-D432.

18. Yin, Y., et al., *dbCAN: a web resource for automated carbohydrate-active enzyme annotation.* Nucleic acids research, 2012. **40**(Web Server issue): p. W445-W451.

19. Team, R., *RStudio: Integrated Development for R. RStudio, Inc.* . 2015.

20. MathWorks, I., *MATLAB*. 2012.

Supplementary Figures and Tables

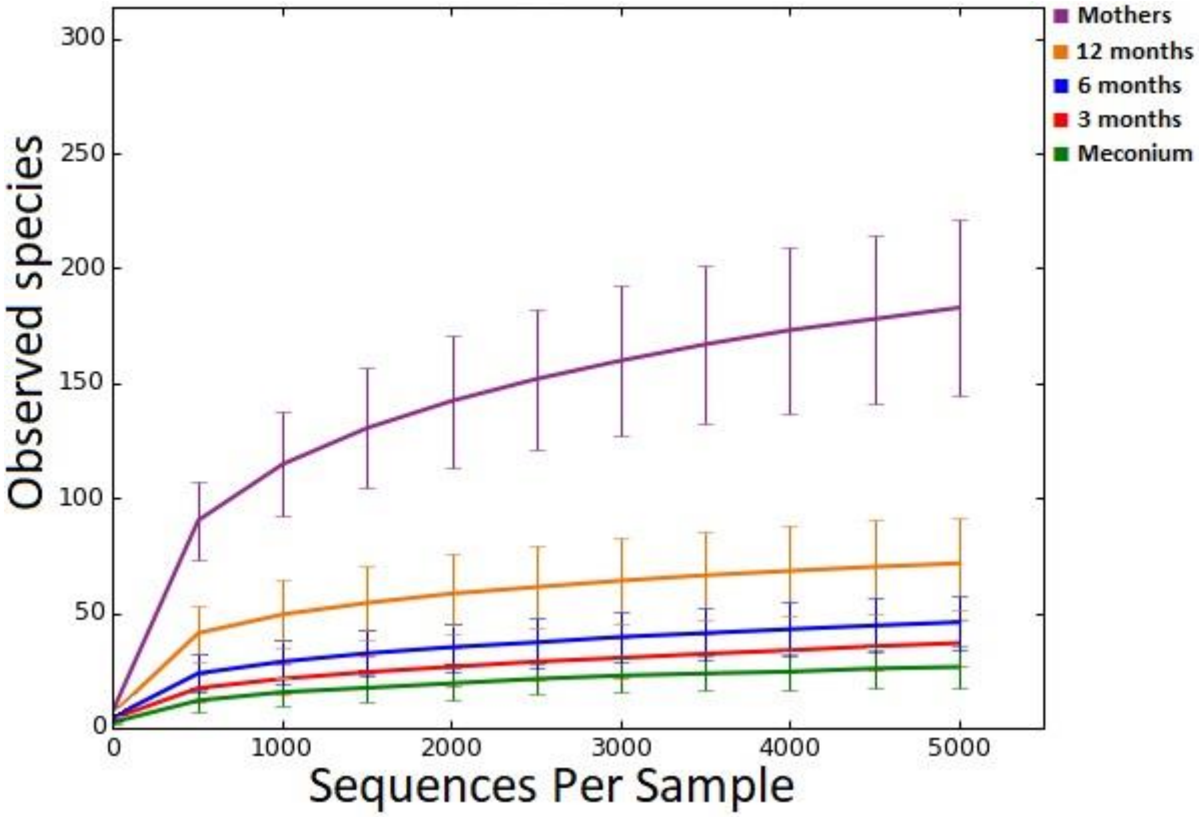

Figure S1. Rarefaction curve of observed species. The illustration shows the amount of observed species based on sequences per sample (SPS) for the different infant age groups and mothers. The cut-off was set to 5 000 SPS. SPS was saturated at approximately 5 000 for infants, but it was not fully established in mothers.

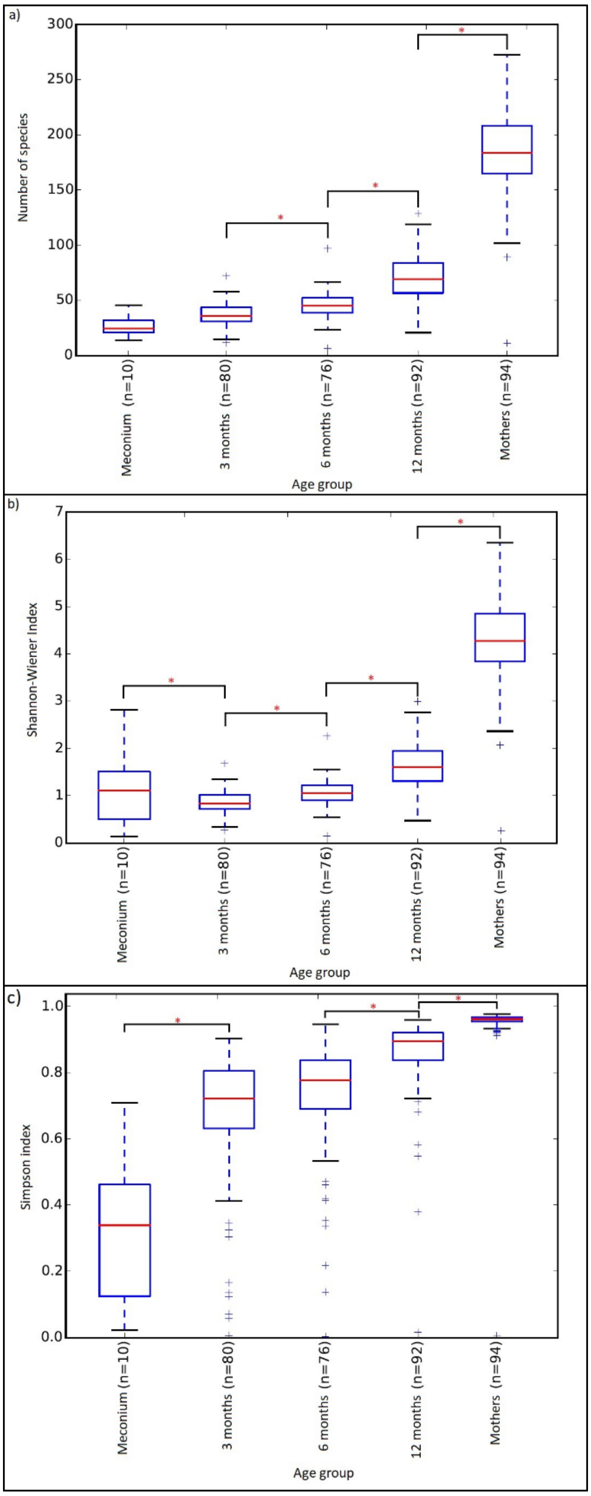

Figure S2. Alpha diversity. The illustration gives an overview of observed species (A), Shannon-Wiener index (B), and inverse Simpsons-index (C). Asterisks represents a p-value < 0.05 (paired t-test).

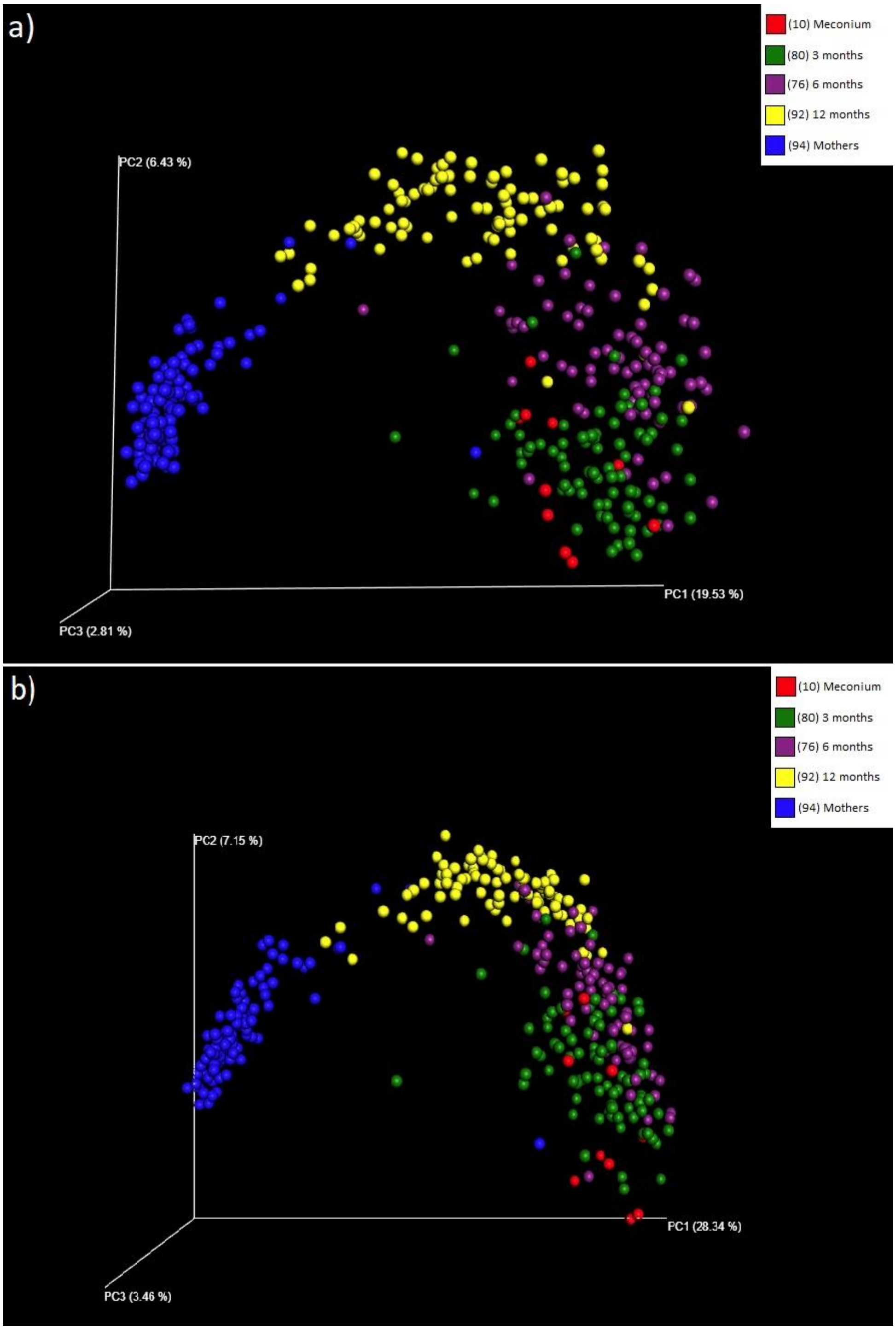

Figure S3. Beta-Diversity. This figure illustrates the Binary-Jaccard (A) and Unweighted Unifrac (B) indexes. The PCoA plot shows the OTU dissimilarities between the different infant age groups and mothers. The age groups are divided by colors, shown at the top right, with the number of samples per age group in parentheses.

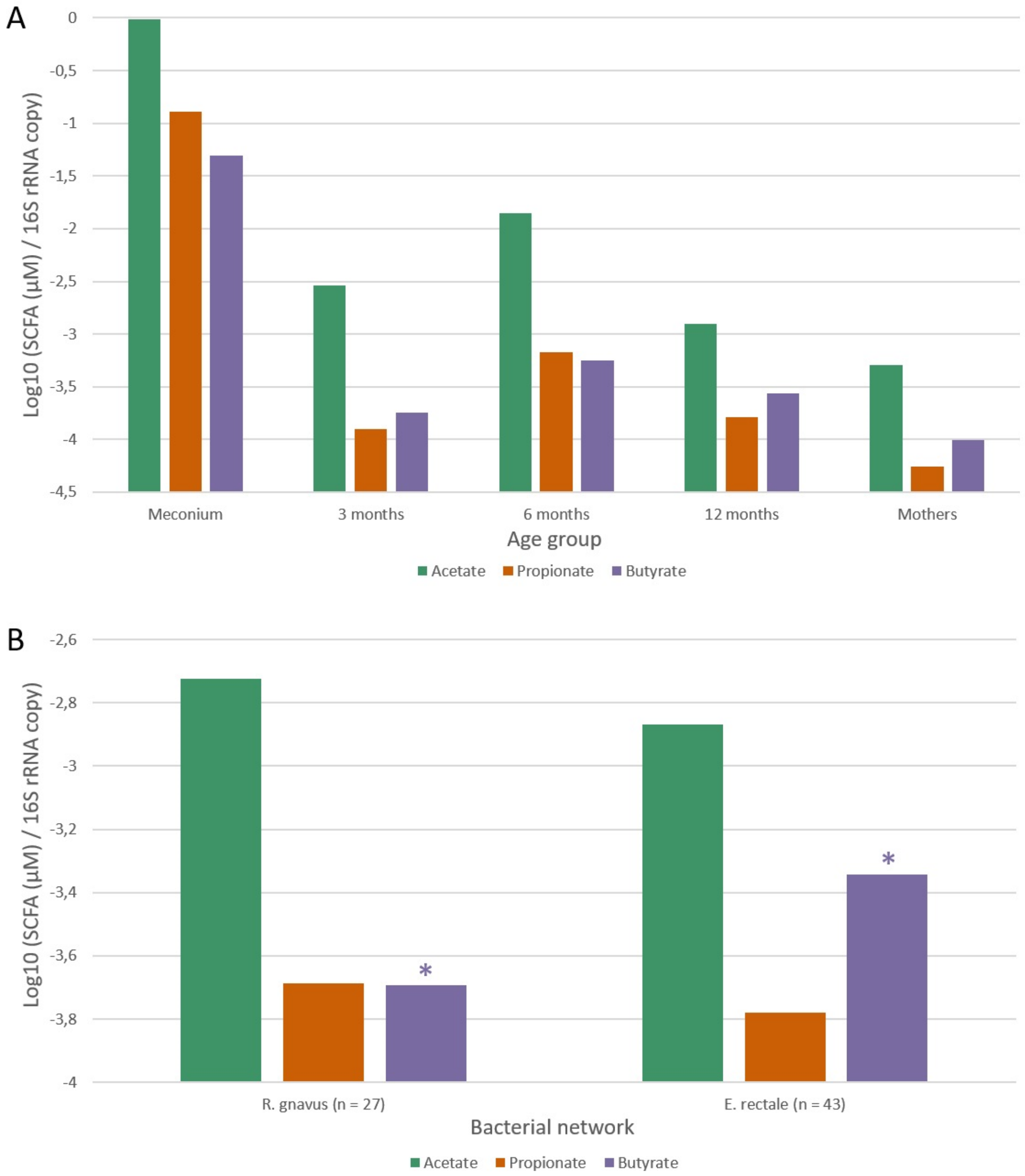

Figure S4. Total short-chain fatty acids per 16S rRNA gene copy. The bar-chart illustrates the logarithmic (log10) abundance of short-chain fatty acids per 16S rRNA gene copy in the infant for all age groups (A) and by the two dominant bacterial networks with a positive or negative correlation to butyrate (B). The bacterial load for each sample was determined by calculating 16S copy number based on Cq-values resulting from qPCR. Asterisks represent a significant difference ($p<0.05$, Mann-Whitney-Wilcoxon test). Asterisks are not included in A) as all SCFAs were significant between the age groups.

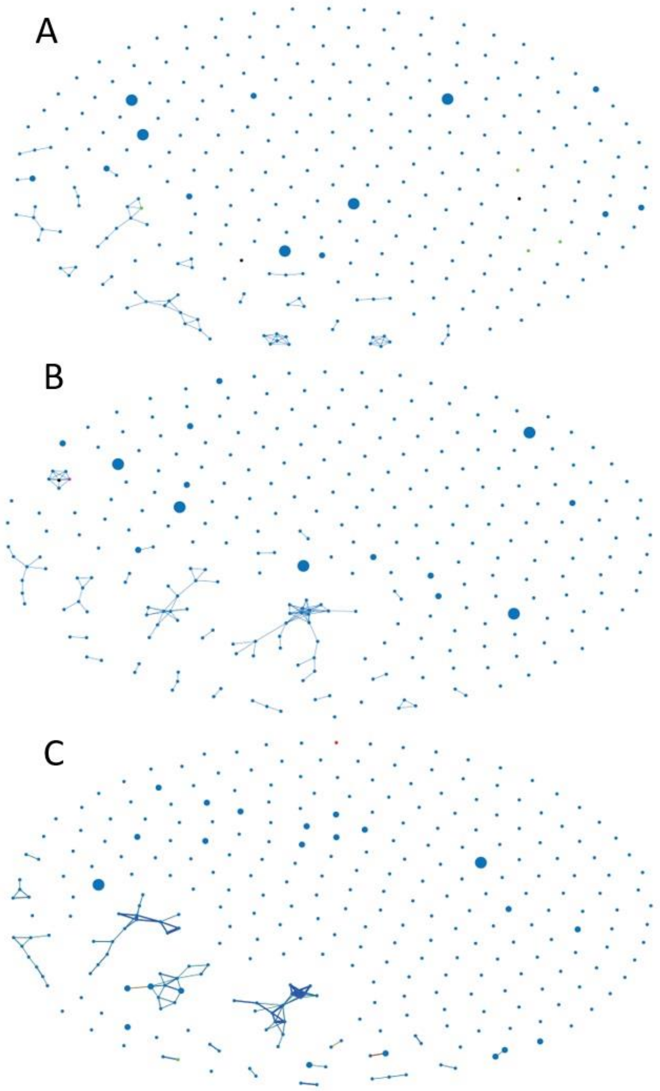

Figure S5. Bacterial correlation to SCFA in all age groups. The figure illustrates the correlation pattern between bacteria and relative amount of SCFAs for 3 months of age (A), 6 months of age (B), and mothers (C). The illustration shows all OTUs from 16S rRNA represented as nodes, with color indicating their correlation to SCFAs; Blue = no correlation, red = negative correlation to butyrate, green = positive correlation to butyrate, black = positive correlation to propionate and purple represents a positive correlation to both propionate and acetate. The three different node sizes represent the general abundance of the respective bacteria. The thickness of the lines between nodes represents a correlation between the bacteria, of which a thick line is a strong correlation. Blue lines indicate a positive correlation between the bacteria, while brown indicate a negative correlation.

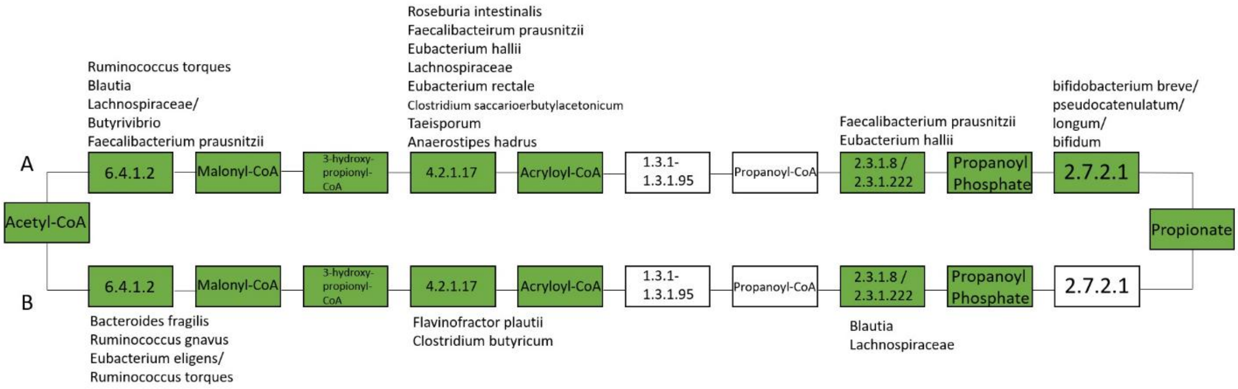

Figure S6. Expressed enzymes related to the propionate production pathway. The figure illustrates the expressed bacterial proteins (represented by E.C numbers) related to propionate production. The figures show proteins detected (green box) in infants with the *E. rectale* network (A), or *R. gnavus* network (B). Bacterial taxonomy is shown next to each E.C number, representing the bacterial source of the given protein.

Table S1. P-values Kruskall-Wallis-Dunn's test for bacterial orders. The table shows p-values for the major bacterial orders between each age group. All values in bold represent a p-value < 0.05.

		**Bacteroidales**		
	**Meconium**	**3 months**	**6 months**	**12 months**
**Meconium**	-	-	-	-
**3 months**	**0.0084**	-	-	-
**6 months**	0.1693	**0.0071**	-	-
**12 months**	0.0622	0.0615	0.2676	-
**Mothers**	**0.0001**	**0.0087**	**4.4×10^{-08}**	**2.4×10^{-06}**
		**Bifidobacteriales**		
	**Meconium**	**3 months**	**6 months**	**12 months**
**Meconium**	-	-	-	-
**3 months**	**3.6×10^{-05}**	-	-	-
**6 months**	**3.6×10^{-05}**	0.963	-	-
**12 months**	0.077	**3.3×10^{-07}**	**4.4×10^{-07}**	-
**Mothers**	0.963	**$<2\times10^{-16}$**	**$<2\times10^{-16}$**	**8.1×10^{-05}**
		**Clostridiales**		
	**Meconium**	**3 months**	**6 months**	**12 months**
**Meconium**	-	-	-	-
**3 months**	0.2271	-	-	-
**6 months**	**0.0135**	**0.0069**	-	-
**12 months**	**1.3×10^{-10}**	**$<2\times10^{-16}$**	**$<2\times10^{-16}$**	-
**Mothers**	**1.5×10^{-08}**	**$<2\times10^{-16}$**	**2.2×10^{-11}**	0.0829
		**Enterobacteriales**		
	**Meconium**	**3 months**	**6 months**	**12 months**
**Meconium**	-	-	-	-
**3 months**	0.9509	-	-	-
**6 months**	0.9509	0.7665	-	-
**12 months**	**0.0037**	**3.3×10^{-10}**	**2.0×10^{-11}**	-
**Mothers**	**1.8×10^{-06}**	**$<2\times10^{-16}$**	**$<2\times10^{-16}$**	**2.5×10^{-05}**
		**Lactobacilliales**		
	**Meconium**	**3 months**	**6 months**	**12 months**
**Meconium**	-	-	-	-

**3 months**	**0.00932**	**-**	**-**	**-**
**6 months**	**0.00337**	0.48377	**-**	**-**
**12 months**	0.41882	**0.00026**	**1.1×10^{-05}**	**-**
**Mothers**	0.62474	**1.0×10^{-11}**	**8.0×10^{-14}**	**0.00190**

Table S2. P-values Kruskall-Wallis Dunn's test for SCFAs. The table shows p-values for the major SCFAs between each age group. All values in bold represent a p-value < 0.05.

	**Acetate**			
	**Meconium**	**3 months**	**6 months**	**12 months**
**Meconium**	-	-	-	-
**3 months**	**0.011**	-	-	-
**6 months**	**0.010**	0.947	-	-
**12 months**	**$<2\times10^{-16}$**	**$<2\times10^{-16}$**	**$<2\times10^{-16}$**	-
**Mothers**	**$<2\times10^{-16}$**	**1.3×10^{-14}**	**1.8×10^{-14}**	0.524
	**Propionate**			
	**Meconium**	**3 months**	**6 months**	**12 months**
**Meconium**	**-**	-	-	-
**3 months**	0.133	-	-	-
**6 months**	**9.7×10^{-05}**	**$4\text{-}9\times10^{-08}$**	-	-
**12 months**	**2.1×10^{-10}**	**4.2×10^{-15}**	**0.017**	-
**Mothers**	**1.9×10^{-10}**	**4.2×10^{-15}**	**0.016**	0.954
	**Butyrate**			
	**Meconium**	**3 months**	**6 months**	**12 months**
**Meconium**	**-**	**-**	**-**	**-**
**3 months**	0.152	**-**	**-**	**-**
**6 months**	**$<2\times10^{-16}$**	**3.8×10^{-16}**	**-**	-
**12 months**	**$<2\times10^{-16}$**	**$<2\times10^{-16}$**	**0.041**	-
**Mothers**	**$<2\times10^{-16}$**	**$<2\times10^{-16}$**	0.654	0.102

Table s3. Maternal and infant factors associated with the presence of either *E. rectale* or the *R. gnavus* network in infants. The table gives an overview of the different metadata: delivery mode, breastfeeding between 3 – 12 months and solid food introduction (3 – 6m) with their respective p-value (chi-square test).

		**Infants with the E. rectale network (n/%)**	**Infants with R. gnavus network (n/%)**	**p-value**
	Vaginal	30/81.1	16/69.6	
**Delivery Mode**	C-Section	7/18.9	6/26.1	.43
	Missing	0/0	1/4.3	
	Boy	21/56.8	9/39.1	
**Gender**	Girl	16/43.2	13/56.5	.66
	Missing	0/0	1/4.3	
	Yes	30/81.1	19/82.61	
**Breastfeeding at 6 months**	No	4/10.81	2/8.7	.81
	Missing	3/8,1	2/8.7	
	Yes	27/73	17/73.9	
**Breastfeeding at 9 months**	No	8/21.6	4/17.4	.75
	Missing	2/5.41	2/8.7	
	Yes	19/51.4	10/43.5	
**Breastfeeding at 12 months**	No	14/37.8	10/43.5	.87
	Missing	4/10.8	3/13.0	
	Yes	2/5.88	4/17.4	.45
**Solids 3m**	No	31/91.2	16/65.6	

	Missing	1/2.94	3/13.0	
	Yes	18/48.65	12/52.2	
**Solids 4m**	No	15/40.54	8/34.8	.36
	Missing	4/10.81	3/13.0	
	Yes	28/75.68	18/78.3	
**Solids 5-6m**	No	5/13.5	2/8.7	.60
	Missing	4/10.81	3/13.0	
